# The Experiences of People From Ethnic Minority Backgrounds Living in Care Homes—A Qualitative Systematic Review

**DOI:** 10.1111/jan.17060

**Published:** 2025-05-19

**Authors:** Lorna Hollowood, Julie Taylor, Kerry Allen

**Affiliations:** ^1^ Department of Nursing and Midwifery, School of Health Sciences, College of Medicine and Health University of Birmingham Birmingham UK; ^2^ Birmingham Women's and Children's Hospital NHS Foundation Trust Birmingham UK; ^3^ School of Social Policy and Society, Health Services Management Centre University of Birmingham Birmingham UK

**Keywords:** ageing, care, cultural issues, death and dying, end of life, ethnicity, literature review, long‐term care, nursing home care, qualitative approaches

## Abstract

The experiences of people from ethnic minority backgrounds living in care homes—A qualitative systematic review.

**Aim:**

Despite the increasing need for older people from ethnic minority backgrounds to be able to access good quality, culturally competent care home provision, globally, there is an absence of literature exploring care home residents' perspectives. This study conducted a systematic review, identifying and synthesising qualitative evidence, which explored the experiences of residents', and their families, from ethnic minority backgrounds, who live in care home settings.

**Design:**

A qualitative systematic review.

Nine electronic databases, MEDLINE, Nursing and Allied Health, CINAHL, ASSIA, AMED, Sociological Abstracts, PsycINFO, Web of Science, SCOPUS, were systematically searched for research published after 2005 until 2025.

**Review Methods:**

This systematic review of qualitative studies was conducted in accordance with The PRISMA 2020 (Preferred Reporting Items for Systematic Reviews and Meta‐analyses) statement. Studies were appraised for quality based upon validated critical appraisal tools from the Joanna Briggs Institute. Qualitative data were extracted and synthesised using reflexive thematic analysis.

**Results:**

Sixteen studies were identified from the international literature that explored care home experiences from the resident's and families' perspectives. Three key themes were extrapolated: Patter, which includes how cross‐cultural communication skills and language affect care experiences; Place, which includes the care home environment, the multi‐ethnic environment, and quality of care; and Person, which encompasses the individual's culture, values, beliefs, food, and family.

**Conclusion:**

There is limited literature from the UK and low‐to‐middle income countries exploring care home residents' perspectives on care provision. Key components of culturally competent care include culturally sensitive communication, adaptable environments that support residents' chosen lifestyles, and inclusive, family‐centred approaches to living well.

**Impact:**

For nurses within the adult social care sector, to recognise the need for further research, education, and policy initiatives aimed at enhancing the care home provision for people from ethnic minority groups.

**Patient or Public Contribution:**

There was no patient or public contribution.

**Summary**

- This review synthesises the existing knowledge and identifies gaps concerning the experiences of ethnic minority individuals residing in care homes.
- It emphasises the importance of culturally competent care, highlighting how factors such as communication barriers, environmental adaptability, and individual cultural needs influence residents' well‐being.
- The findings advocate for the development of effective strategies and policies to enhance culturally responsive care practices within long term care settings as well as more primary research which explores the resident's perspectives.

## 1 Introduction

The increasing, ageing population worldwide challenges policymakers and long‐term care providers. The World Health Organization (WHO) [2017a] predicts that one in five will be over 60 by 2050. Globalisation and increased international migration mean that there are more people from ethnic minority backgrounds living and ageing in different countries than ever before (United Nations 2020; International Organization for Migration 2020). Health and social care providers need to review their provision of services to ensure the ability to meet the needs of elders from ethnic minority communities. Global estimates suggest that 10% of people over the age of 65 have long‐term care needs and up to 68% have those needs met in a care home (OECD 2021). There are huge variations in access to long‐term care with increasing numbers of people living at the end of their lives in long‐term settings in high‐income countries and a significant lack of services in low‐and middle‐income countries (Broad et al. 2013; WHO 2017b). Access to good quality care homes is a global priority in the Decade of Health Ageing strategy (WHO 2020), and this drives the need to improve care provision by understanding the experiences of care home residents and their families (Bradshaw et al. 2012). With almost 50,000 nurses currently employed in the sector in the UK, there are implications for policy, education and research in this area to ensure an informed and responsive workforce (Skills for Care 2024).

Accurately estimating the number of individuals from ethnic minority backgrounds residing in care homes, globally, is challenging due to inadequate and inconsistent recording of ethnicity in healthcare systems (Badger et al. 2009). The COVID‐19 pandemic has further exposed vulnerabilities within the sector, shedding light on critical issues such as workforce shortages, limited access to broader healthcare services, and gaps in regulatory oversight (Davidson and Szanton 2020). In England, data from the Care Quality Commission (2022) revealed a disproportionate number of deaths among Black and ethnic minority individuals in care homes during the pandemic. While the lack of comprehensive ethnicity data limits the contextualisation of these findings, they align with existing evidence on the profound health inequalities faced by older adults from ethnic minority backgrounds (Stopforth et al. 2023). These inequalities, often rooted in cumulative disadvantage and sustained racial discrimination, result in poorer health outcomes in later life (Stopforth et al. 2023).

There is a rising need for care home provision globally, which has financial implications for both society and individuals, and this contributes to the rationale of this systematic review in seeking the resident's perspective (Armstrong 2018). Research exploring the needs of older people from ethnic minority backgrounds in long‐term care settings remains limited, and there are several factors contributing to this. We found no UK‐based research and a complete absence of research from low and middle‐income countries. Lillekroken et al.'s (2023) systematic review of family caregiver's experiences, identified several studies that highlighted a preference for informal caregiving across certain ethnic minority groups. This could be a contributory factor for the underrepresentation of some groups within care home settings, certainly in the UK where the care home population is 97.5% made up of individuals identifying as ‘White’ ethnic group in the most recent census (Office for National Statistics 2023). There is a need for more focussed research on how certain minoritised groups access care as they may experience additional barriers which contribute to the underrepresentation. Gove et al. (2021) identified language barriers, cultural stigma and general distrust as inhibiting factors for access. The study also goes on to explore differences in health literacy and structural discrimination in existing services as obstacles. A recent systematic review identified similar barriers hindering access to care homes for minority populations, including inappropriate language use, fear of discrimination, and familial expectations (Scott et al. 2022). Cabote et al.'s (2022) integrative review recognises that existing studies do not adequately consider the needs of older adults with culturally and linguistically diverse backgrounds and this can potentially impact on provision of tailored care. An earlier review in 2005 identified a paucity of research in the UK that examined the needs of ethnic minority care home residents (Mold et al. 2005). Mold et al. (2005) identified an urgent need to conduct more research to understand the impact of care on the quality of life to deliver high‐quality services. Despite these recommendations, there is still an absence of empirical studies exploring the needs of this vulnerable and seemingly voiceless group. The lack of research led to this review becoming international in its breadth and informed the search dates of those published after 2004. More empirical studies, that seek to understand the experiences and the care needs of those from ethnic minority backgrounds, such as Xiao et al. (2023), will support the critical gap in our understanding of those needs and how understanding will support the journey to culturally competent care in these settings. Addressing this gap is essential for informing culturally competent care practices and ensuring equitable care for diverse older populations.

## 2 Aims

The purpose of this review was to synthesise qualitative studies that examine experiences of residents from ethnic minority backgrounds and their families, who live in care home settings. The decision to focus on qualitative studies in this review was driven by the need to explore the context‐specific experiences of ethnic minority residents, which are not captured by qualitative measures. In‐depth insights into the personal experiences of residents were sought to better understand the unique challenges they face, in relation to cultural identity.

The research questions were.

For people from an ethnic minority background.

1. What are the experiences of living in a care home setting?
2. What are the experiences of their caregivers and family members?

### 2.1 Additional Enquiry Includes

- What are the components of culturally competent care in a care home?
- What are the key needs of people from ethnic minority backgrounds living in a care home setting?

## 3 Methods

### 3.1 Design

This systematic review of qualitative studies was conducted in accordance with The PRISMA 2020 (Preferred Reporting Items for Systematic Reviews and Meta‐analyses) statement (Page et al. 2020). All studies included for review were qualitative that collected data from care home residents and their family members and caregivers. Due to limited resources, only studies published in the English language were included.

### 3.2 Protocol and Registration

A protocol was designed adhering to the PRISMA‐P guidelines (Moher et al. 2015). This was registered with Prospero International Prospective Register of Systematic Reviews related to health care and social sciences (registration number: CRD42020198639) and can be accessed on the Prospero website (https://www.crd.york.ac.uk/prospero).

### 3.3 Search Strategy

A search of nine electronic databases (MEDLINE, Nursing and Allied Health, CINAHL, ASSIA, AMED, Sociological Abstracts, PsycINFO, Web of Science, and SCOPUS) was undertaken, followed by a manual search of references. Searches were originally undertaken from January 2023 to June 2023 and updated in January 2025 for relevant publications. The updated search yielded an additional 2 papers for inclusion. The wide range of databases was chosen to reflect the diversity of needs that the population group covers and to recognise the range of disciplines spanning both health and social sciences. The search start date of 2005 was chosen, as it aligns with the publication of a key UK‐based literature review by Mold et al. (2005), which offers an early exploration of this topic and provides a foundational benchmark for subsequent research developments. Search terms included nursing home; care home; residential home or long‐term care; ethnic*, race, minor*, cultural diversity; cultural and linguistic diversity (CALD); residents or patients. The search strategy was supported in development by specialist academic librarians. (Example of search strategy in File S2).

### 3.4 Inclusion and Exclusion Criteria

The phenomena of interest for our review were data relating to the experiences of those from ethnic minority backgrounds living in care homes. This included family and primary caregiver's perspectives. Studies were included if they met the following inclusion criteria: (i) primary study using qualitative methodology; (ii) a focus on residents' and their families’' experiences of care home settings, who from ethnic minority backgrounds; (iii) focusing on older adults; and (iv) published post 2005 and in English Exclusion criteria included studies the following: (i) conducted in community or hospital settings; (ii) focused on staff experiences; and (iii) not specific to residents from ethnic minority backgrounds.

### 3.5 Data Collection

To reduce bias over inclusion and exclusion decisions, results were uploaded to systematic review screening software, RAYYAN (https://rayyan.qcri.org/), allowing a transparent screening process between the research team and facilitating greater collaboration (Johnson and Phillips 2018). One researcher conducted the searches and updated searches, and two co‐researchers supported the screening selection process. This allowed for discussion of discrepancies and added rigour to the selection process. Data extraction has been standardised by using a table to ensure relevant information was captured, providing consistency and enhancing validity and reliability (NHS Centre for Reviews and Dissemination 2009) (Table 1).

**TABLE 1:** Data abstraction.

Author, year of publication and country of origin	Aim	Sample size	Sample	Design/data collection	Data analysis	Key findings and conclusions	Limitations
Caldwell et al. (2014) Australia	To investigate the decision‐making process for placing a person with dementia on a waiting list for a nursing home and the influence of cultural factors, comparing caregivers from Chinese and English‐speaking backgrounds	27	Caregivers of people living with dementia from Chinese backgrounds (20) and English‐speaking backgrounds (7)	Qualitative Semi‐structured interviews	Thematic analysis (Braun and Clarke) NVivo software used for coding	When caregivers apply for a waiting list, some were ready for placement, others “just in case,” and for some there was no waiting time because of an urgent need for placement Reasons why caregivers apply—emotions, expectations of nursing homes. The decision‐making process was similar for both cultural groups, but Chinese caregivers spoke more about their sense of duty, the need for a Chinese specific facility, and declining a place because of family disagreement Chinese caregivers had additional considerations regarding nursing homes, food and language, English‐speaking backgrounds, and had home location (near) as a stronger consideration	Small number of English‐speaking background participants Potential sampling bias—only 10% response in English‐speaking background group Potential for recall bias as some caregivers had made the decision 12 months ago Caregiver satisfaction may have influenced decision to participate
Chan and Kayser‐Jones (2005) US	To investigate the clinical, environmental, social, and cultural factors influencing provision of end of life care	34	Chinese nursing home residents	Qualitative data were obtained through participant observation, event analysis, and in‐depth interviews with residents and their families, nursing staff, and physicians	Event analysis	The most significant factors influencing the care Chinese residents received were: Communication barriers—79% of participants were unable to speak English, meaning it was difficult to express needs or socialise Dislike of Western food leading to weight loss and reliance on family food provision Differing cultural beliefs and customs around use of traditional chinese medicine, taboo around talking about death and Buddhist spiritual beliefs and end of life practices	Findings are not generalisable due to being set in only two nursing homes
Girard and El Mabchour (2019) Quebec, Canada	To gain a better understanding of the meal context and the food offering in Quebec public nursing homes for non‐autonomous seniors, particularly with respect to first‐generation immigrants	101	Non‐Quebec‐born residents (*n* = 26), their families (*n* = 24) and frontline care staff (*n* = 51)	Qualitative—A focused ethnography approach. Semi‐structured interviews and structured non‐participative observations were made in facilities	Thematic analysis and coding of transcripts	Residents who are first generation immigrants adapted with difficulty and often not at all to the food offering. Residents' appetite for food offer was a problem for reasons related primarily to food quality, mealtime schedules, medication intake, physical and mental condition, and adaptation to institutional life. Family/friends often brought in food	A degree of heterogeneity in the focus groups with staff. Also, the presence in a few cases of a hierarchical superior may have influenced the discourse of the staff in these groups Scope of the study limited to residents' experiences with food and may have missed important narratives about the immigrant journey. Similarly staff roles and worthiness were not explored
Hefele et al. (2016) US	What kinds of information do consumers want and need when making a nursing home selection? Do these preferences vary across racial/ethnic groups?	105	White, black, and Latino adults aged 65+ and 40–64 years, who had personal/familial experience with a NH admission or concerns about one	Qualitative—Eleven focus groups and 30 interviews were conducted separately by race/ethnicity and age group	Systematic coding to generate themes to each racial group	Participants wanted detailed information on the facility, policies, staff, and residents, such as location, staff treatment of residents, and resident conditions. They wanted a sense of the NH gestalt and were interested in feedback/reviews from residents/families. Black and Latino participants were especially interested in resident and staff racial/ethnic concordance and facility cultural sensitivity. Latino participants wanted information on staff and resident language concordance	The sample was limited to the greater Boston area, and we did not use a random sample to select participants for this study. Limited generalisability and findings must be understood within this context, including that the results may be biased towards an urban population. Three Racial groups are explored—cannot assume heterogeneity in any one racial group
Heikkilä et al. (2007) Sweden	To describe one model of a culturally congruent care setting for older people, the Finnish Home in Sweden, and how cultural congruency is used in care for older Finnish immigrants in order to promote their well‐being	Ethnological study in nursing home with 50 beds. Incl 20 interviews	20 nursing staff, residents and their visitors	Ethnological study over 13 months Spontaneous interviews of 40–70 min	Hermeneutical approach to analysis	Cultural congruency, based on the residents' mother language, a shared ethnic background with the staff, and shared customs, creates a common ground for communication and understanding. This enables caring relationships, which in turn increases the residents' well‐being	The results concentrate on one particular context
Hutchinson et al. (2011) US	To investigate person and environment factors of elders that facilitate adaptation to relocation to long‐term care skilled nursing facilities	23	Newly admitted Caucasian and African American nursing home residents. Purposive sampling used	Qualitative interviews using a phenomenological approach	NVivo transcribing software and phenomenological analysis	Themes that emerged include (a) spirituality, death and dying, and philosophy of life; (b) life experiences with change; (c) cultural heritage; (d) health; (e) ethnicity; (f) social support, family and friends; (g) long‐term care facility (LTCF) relationships; (h) LTCF system maintenance; and (i) LTCF support of personal growth. Comparison of African Americans and Caucasians showed more similarities than differences between the groups	The study only explored the views of two ethnic groups
Koehn et al. (2018) Canada	Explores the extent to which family members who regularly visit Cantonese‐speaking residents in two LTRC facilities, understand and utilise Family Councils Explicate how the residents and family carers interviewed perceived quality of care and quality of life and if engagement with family councils had an impact on that	20	Purposive sample of nine Chinese‐origin residents living in LTRC homes and 11 family carers	Qualitative case study approach included participant observation and individual interviews	Thematic analysis using a critical gerontology and intersectionality framework	Participation in Family Councils—thought it was important to have them but very few participated thinking their views were not welcome Language barriers impacted on quality of care, care costs deemed appropriate, level of training impacted views of carers that is, nurses and nutritionists held in higher regard. Communication was the biggest issue Quality of life—social environment deemed most important, opportunity to socialise and play games with other Cantonese residents. Food was source of complaint as not Cantonese. Loss of decision‐making capacity and freedom an issue for residents	The identity of the interviewer, a young Chinese male, inevitably played a role in the co‐construction of the data with our participants (Denzin 1994). This bias is offset to some degree by his skill as an interviewer and translator. Small sample size results in lack of generalisability
Kong et al. (2010) US	To describe Korean immigrant caregivers' experiences regarding American nursing home placement of their non‐English‐speaking older relatives with dementia	10	10 Korean immigrant family caregivers	Qualitative descriptive methods using semi‐structured interviews	Qualitative content analysis	The “Korean way of thinking” emerged as a fundamental cultural belief about caregiving. Six major themes were identified: (a) I never thought about a nursing home; (b) If I think in a Korean way, I feel …; (c) Nursing home staff cannot communicate with …; (d) My care recipient maintains Korean culture; (e) Nursing home services are better than expected but …; and (f) My care recipient is more vulnerable because of dementia. This study provides valuable insights for health care providers about communication and cultural issues of immigrant caregivers for non‐English‐speaking older relatives with dementia	Sampling bias is a limitation of the study in terms of the region for recruitment, transferability may be limited
Ott (2008) US	To identify why African American nursing home residents had not completed a living will	28	African American nursing home residents purposively sampled from three nursing homes	Qualitative—Exploratory study using focus groups	Coded and thematically analysed	Five themes emerged The value of artificial life supporting treatments Communication about end‐of‐life preferences. Involvement of family members in end‐of‐life decisions Physician involvement in end‐of‐life decisions The value of a living will	A small study conducted in a specific geographic location in nursing homes selected by the researcher
Park et al. (2013) US	To explore resident‐to‐resident and resident‐to‐staff relationships experienced and perceived by African American and Hispanic older residents	30	15 African American and 15 Hispanic older adults in seven assisted living communities in Central Florida	Qualitative—in‐depth interviews	Grounded theory—thematic analysis	The major themes of the study are formation of relationships, language as a facilitator or barrier, and avoidance of inter‐racial/ethnic relationships. When language and culture can be shared, cultural values and historical experiences of African American and Hispanic older adults should be considered in daily practice in assisted living facilities. The assisted living communities should provide culturally competent social environments for residents and staff Food is also a quality of life issue	Small number of participants in a specific geographical location. Not all relationships (interracial) explored
Thao et al. (2025) US	The aim of the study is to examine the quality of life (QOL) experiences of Hmong older adults in nursing homes (1) Investigating how language barriers, race, and ethnicity combine to impact QOL for Hmong NH residents (2) Exploring how these factors may contribute to social isolation, neglect, and dissatisfaction with food and activities among Hmong residents	19	Eight Hmong residents, five Hmong staff, and six participants in focus group (including family and community members)	Qualitative case study design. Data collected by individual interviews, observations over a 4 month period and community focus group	Thematic analysis	The key finding was that Hmong residents experience a diminished QoL in nursing homes due to the following factors Neglected care needs, limited relationships and isolation, limited activities, lack of food enjoyment and ‘like a caged pig’—residents felt they just live and wait to eat	Limited generalisability. Focus on one ethnic group Lack of specific measure of limited English proficiency (which limits comparison in differences of QoL)
Xiao et al. (2023) Australia	The aim of this study was to compare factors affecting residents' ability to fulfil self‐determination in ethno‐specific and mainstream care homes. The study sought to understand how different care environments impact residents' autonomy, competence, and ability to maintain meaningful relationships	29	Twenty‐four residents and five family members	Qualitative descriptive approach with in‐depth interviews and one focus group	Thematic analysis	Three main themes of factors which contribute to resident's self‐determination (1) Communicating needs and preferences (2) Mastering own care and (3) Maintaining meaningful relationships. Culturally competent and person‐centred care are fundamental for residents' self‐determination and that families play a critical role, ethno‐specific homes provide better support for social interactions through religious activities, and residents desire more opportunities to connect with peers	The study's limitations include limited representation of ethnic minorities in mainstream nursing homes, limited representation of the mainstream culture in ethno‐specific nursing homes, and a low number of family member participants, which may affect the generalisability of the findings
Xiao et al. (2017) Australia	This study explores (i) residents' and family members' perceptions about staff and cultural diversity, and (ii) culturally and linguistically diverse residents' and family members' experiences	30	Twenty‐three residents and seven family members	Qualitative—Convenience sample from four aged care homes, participants interviewed	Interpretative design, thematic analysis Demographic data statistically analysed (SPSS)	Four themes identified (i) perceiving diversity as an attraction; (ii) adapting to cross‐cultural communication; (iii) adjusting to diet in the residential care home; and (iv) anticipating individualised psychosocial interactions. The findings have implications for identifying strategies to support staff from all cultural backgrounds in order to create a caring environment that facilitates positive relationships with residents and supports residents to adjust to the care home	Use of interviews limited the researchers' ability to. explore how residents with dementia adjusted to the diverse care settings and their responses to cross‐cultural communication. Field observations may have revealed more. Low number of CALD residents willing to be interviewed
Xiao et al. (2018) Australia	The aim of this study was to critically examine how staff and residents initiated effective cross‐ cultural communication and social cohesion that enabled positive changes to occur	86	Participants included 23 residents, seven family members and 56 staff	Qualitative—One to one interviews with residents and focus groups with staff	Double hermeneutic design, interpretative, critical theory, and thematic analysis	Four themes were identified as social conditions that enable resident–staff CCC. These themes were as follows: (i) restructuring communication via a partnership approach; (ii) co‐developing communication resources; (iii) empowering CALD residents in communication; and (iv) improving residents' experiences in cross‐cultural care through a systematic approach Staff and residents showed a reciprocal role in enhancing cross‐cultural communication and through demonstrating mutual cultural humility	Small numbers of staff and residents The mainly focused on how linguistic diversity affected CCC. More studies are needed to explore the intersection of both cultural and linguistic diversity on CCC The study used interviews and focus groups that might not capture the interplay of actions and structures in real situations
Yeboah et al. (2013) Australia	To identify the experiences of culturally and linguistically diverse (CALD) older adults of relocating into residential homes and their post relocation experiences	12	Two women and eight men. All were born overseas: Italy (5); Greece (3); Macedonia (2); Croatia (3); Latvia (1); Malta (2); Russia (1); and India (3)	Qualitative, using grounded theory methodology, interviews were conducted with 20 residents at four nursing homes	Continuous process, consistent with grounded theory method (GTM) data analysis, and consisted of initial open coding, axial coding and final selective coding. Data were analysed using the constant comparative method	Seven components of the pathway to aged care were identified in this study: (1) experiencing losses; (2) responding to losses; (3) considering offers of care; (4) deciding to move to residential aged care; (5) searching for and selecting a nursing home; (6) moving to a nursing home; and (7) settling in	Participants limited to CALD older people in only four nursing homes. More women due to women living longer. Only able to recruit fluent English speakers
Yeboah (2015) Australia	This paper discusses the cultural underpinnings identified by CALD nursing home residents during their relocation decision‐making	12	Two women and eight men. All were born overseas: Italy (5); Greece (3); Macedonia (2); Croatia (3); Latvia (1); Malta (2); Russia (1); and India (3)	Qualitative, using grounded theory methodology, interviews were conducted with 20 residents at four nursing homes	Continuous process, consistent with grounded theory method (GTM) data analysis, and consisted of initial open coding, axial coding and final selective coding. Data were analysed using the constant comparative method	From early in the analysis, participants described reciprocity as being core to their relocation experiences and fundamental to maintaining cultural connections during and following the relocation. Significantly, reciprocity was identified by many of the participants from different cultures as reflecting and reconfirming their culture. Participants in this study described three types of generalised reciprocity: historical, concurrent, and individual It is the feeling of consistency with one's own culture that provided the sense of cultural continuity	Participants limited to CALD older people in only 4 nursing homes. More women due to women living longer. Only able to recruit fluent English speakers. There is the possibility that the experience of other elderly CALD people (e.g., Africans) may differ from other populations or cultural groups participating in the study

### 3.6 Quality Appraisal

All studies were appraised individually for quality based upon validated critical appraisal tools from the Joanna Briggs Institute (2020) and then assessed for quality and rigour. Each study was assigned a three‐point scale system taking into consideration research design, methods, and rigour (Taylor et al. 2012). A quality score of 1 demonstrates confidence in the research design, rigour, and bias addressed adequately, 2 for satisfaction with design and attempts to address bias, and 3 for studies that demonstrated design flaws, poor attempts to address bias, or not satisfying a basic criterion for rigour (Taylor et al. 2012). A three‐point scale was also used to establish the usefulness of each paper in addressing the review objectives. A score of 1 was given to papers which directly answered the review objectives and were deemed extremely useful, 2 to papers which provided useful insight into the experiences of those in long‐term care settings, and 3 to those which added very little to the evidence base in this field (Taylor et al. 2012). This process resulted in the omission of one paper from the results due to a lack of usefulness to the review's objective. (Quality and Usefulness Table in File S1).

### 3.7 Data Synthesis

Braun and Clarke's (2022) approach to reflexive thematic analysis, was used to synthesise the data to develop overarching themes. Thematic analysis was chosen due to its suitability to explore the unique and rich lived experiences of ethnic minority residents in care homes. This method allowed some flexibility to capture the broader patterns that emerged within the thematic analysis but also provided an opportunity to capture some of the individual experiences of participants within the studies. In line with the principles of reflexive thematic analysis, the process began with the extraction of data from the primary studies, focusing on verbatim quotes from participants to ensure the authenticity of their voices. These quotes were then coded into initial meaningful units, capturing single ideas or concepts directly related to the research questions (example of thematic analysis in File S3). Using a visual mapping technique, three themes around key areas of experience were developed (Braun and Clark 2022). The primary authors' interpretations in the studies were also considered, but the emphasis was placed on the participants' narratives to maintain a focus on lived experiences.

## 4 Results

**FIGURE 1:**
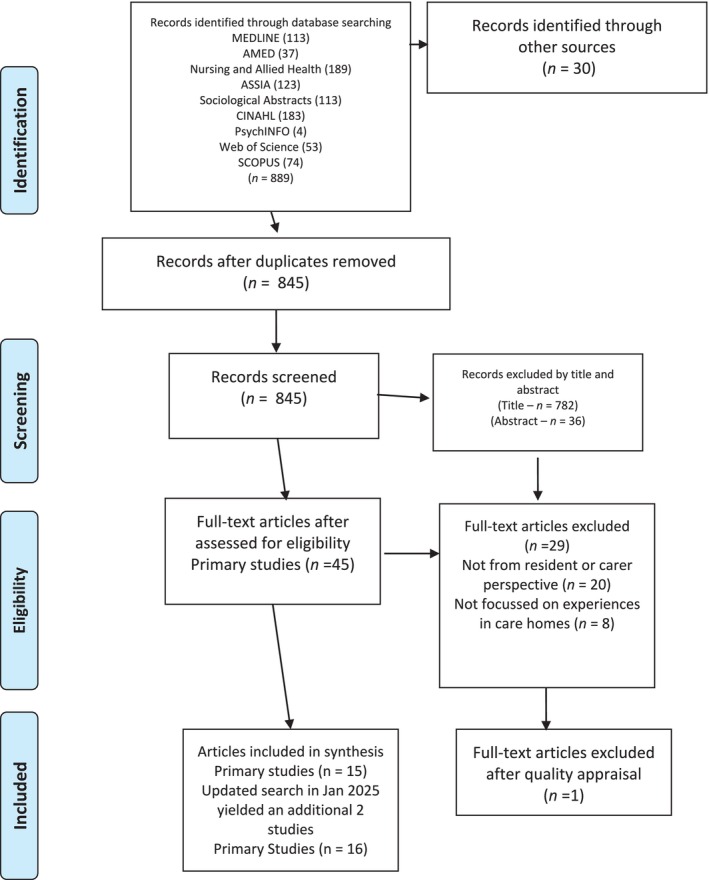
PRISMA for systematic review.

A total of 888 studies were identified, and of these, 45 full texts were retrieved and reviewed independently by three reviewers according to the inclusion and exclusion criteria. In total, 16 papers were deemed eligible for synthesis. While some included studies encompassed perspectives from healthcare staff, our analysis was confined solely to data derived from residents and their family members to maintain focus on the primary phenomena of interest. The studies were published between 2005 and 2025, all from studies conducted in high‐income countries: US (n7), Australia (n6), Canada (n2) and Sweden (n1). The search results are shown in Figure 1.

### 4.1 Findings

The findings of this review are presented in three themes: *Patter*, which includes how cross‐cultural communication skills and language affect care experiences; Place, which includes the care home environment, the multi‐ethnic environment and quality of care; and *Person*, which encompasses the individual's culture, values, beliefs, food and family.

### 4.2 Theme 1: Patter

#### 4.2.1 Communication and Language

Challenges around communication and languages spoken were identified in 12 of the studies (Caldwell et al. 2014; Chan and Kayser‐Jones 2005; Hefele et al. 2016; Heikkilä et al. 2007; Koehn et al. 2018; Kong et al. 2010; Park et al. 2013; Thao et al. 2025; Xiao et al. 2017, 2018, 2023; Yeboah et al. 2013). Access to bilingual care providers was identified as a barrier to accessing care homes (Caldwell et al. 2014) and as a factor in the poor wellbeing of residents (Chan and Kayser‐Jones 2005; Kong et al. 2010; Thao et al. 2025). This was important for staff–resident relationships and residents' peer relationships within the homes and impacts on social isolation levels and quality of life (Hefele et al. 2016; Heikkilä et al. 2007; Koehn et al. 2018; Park et al. 2013; Thao et al. 2025; Xiao et al. 2023).

English is the main issue. When there is no one to translate, the misunderstanding is huge! It's like chickens talking to ducks [Chinese proverb] (C02). (Koehn et al. 2018, 169)

They [Hispanic residents] are good people. They care for me, they talk to me, and we have conversations that I enjoy. We are friends and we love each other very much. … We share conversations and we understand each other's language and we love each other. (Park et al. 2013, 381)

Care home staff having the skills and knowledge to deliver communication in a culturally appropriate way has a positive impact on the resident's wellbeing (Haesook et al. 2014; Xiao et al. 2023). A facilitator to effective relationships within care homes was found in an Australian study that saw staff, residents, and families collaborating on developing cross‐cultural communication resources, such as phrase books as translation aids (Xiao et al. 2018).

… often when we have someone from a different background the families will put together a booklet of basic things, like ‘Would you like a cup of tea,’ or ‘Are you in pain?’ Along with a picture book—yeah, it's a book of a translation that we can then refer to them (S1). (Xiao et al. 2018, 7)

Participants identified differences in communication methods, such as interpretation issues, cultural misappropriation and a lack of understanding as having a negative impact on care provision and contributing to neglectful care practices (Xiao et al. 2017; Kong et al. 2010; Thao et al. 2025; Yeboah 2015).

Effective communication was enhanced for many participants across the study when there were others from similar ethnic backgrounds among the staff and residents in a home (Caldwell et al. 2014; Hefele et al. 2016; Heikkilä et al. 2007; Park et al. 2013; Thao et al. 2025; Xiao et al. 2017, 2023).

… that would make a difference, wanting to make sure that there were people there that look like the resident I was bringing (OB participant). (Hefele et al. 2016, 1176)

Yes [relationships to staff are important] because I speak more because they [staff] speak Spanish. … This place's residents don't speak Spanish, but with them [staff] … they always, always look for me. (Park et al. 2013, 381)

But there is not many of us that can say that we speak English especially when it is necessary. When it is necessary, we find somebody here to translate. (Xiao et al. 2023, 6)

Acknowledging different cultural beliefs and satisfaction with care was enhanced by a multi‐ethnic environment (Chan and Kayser‐Jones 2005; Xiao et al. 2017, 2023; Thao et al. 2025). One study identified that when cross‐cultural communication issues were observed and addressed by both residents and staff from differing backgrounds, this led to the empowerment of both groups, and an environment of cultural safety and humility was fostered (Xiao et al. 2018, 2023).

A multi‐ethnic environment is not solely a positive experience for all care home residents though, and this was explored by Park et al. (2013), who unearthed racial tension in their study in a multi‐ethnic care facility that included White, Hispanic, and Black residents.

I try to ignore certain things around here. They [the residents] are talking race, you know. … There is a White lady. She could've been White or Puerto Rican. But she look[s] White, and she was talking about these niggers this and these niggers that. And I was sitting, you know, a chair away from her and she knew where I was sitting because she had been sitting across from me. But she was talking about somebody else, so I guess she figured her talking about somebody else. I shouldn't, you know, have, be affected of her talking, you know, race … [but] it made me madder than hell, you know? … (African American woman in her early 60s) (Park et al. 2013, 383)

### 4.3 Theme 2: Place

#### 4.3.1 Experiences of Care

Quality of care and how staff treat residents was an issue for participants across the literature, and information was identified as a prerequisite for admission.

It is very important that they are sensitive to our culture. … … … That happens and I don't know why, and you feel discriminated by the way they talk to you or by certain attitudes. (Hefele et al. 2016, 1179)

… that the staff is kind and gentle with the patients, because sometimes these patients can be obnoxious. (Hefele et al. 2016, 1175)

Participants identified differences in cultural factors having an impact on care provision often due to misunderstandings. These include interpretation due to language difficulties, cultural misappropriation, a lack of understanding and service design for mainstream culture (Kong et al. 2010; Xiao et al. 2017; Yeboah 2015). Chan and Kayser‐Jones (2005) noted that participants had cultural reasons which underpinned aspects of care that they did not want to discuss, for example, decisions around resuscitation but that led to a fear of suffering if active treatment was ‘withdrawn’. These differences and misunderstandings have the potential to lead to difficulties in care delivery and personal care can be a point of tension. A family member describes her mother as hitting out at care staff during personal care because she did not like taking her clothes off in front of people (Kong et al. 2010).

Mrs. Hahn stated that her mother resisted taking off her clothing and exposing her naked body to others, which resulted in her mother's hitting a nurse aide. Mrs. Hahn's mother was sent to a psychiatric hospital because she hit nurse aides during bathing time. She attributed her mother's dislike of taking off clothing in front of other people to Korean tradition rather than a violent nature. In traditional Korean culture, exposing the body to other people was not acceptable, especially for women. (Kong et al. 2010, 325)

Participants in one study, exploring the quality of life experiences of Hmong older adults in a nursing home, identified a difference in care provision dependant on ethnicity, noting that white residents received personal care more regularly. A Hmong resident who shared a room with a white resident stated her roommate had her clothes changed more frequently.

it comes to you (Hmong NH residents), they don't even care. (Thao et al. 2025, 269)

Three studies linked care provision to quality of life and identified that participants from ethnic minority backgrounds had unmet psychosocial needs, reduced social interaction and lower social engagement than those from the mainstream culture within the setting (Xiao et al. 2017, 2023; Thao et al. 2025). These findings were clearly underpinned by the staff in the care settings, practice patterns, resources and policies.

#### 4.3.2 End of Life Care

Good quality end of life care in the care home setting includes residents being identified as being in the last years of life, engaged in planning of care including Advance Care Planning discussions, good assessment and treatment of symptoms at end of life, and death occurring in the preferred place of care (Shaw et al. 2010; Badger et al. 2009).

Collaboration between staff and residents and their families contributed to perceptions around good care around end of life issues, with participants expressing a fear of palliative care and withdrawal of active treatment (Chan and Kayser‐Jones 2005). A cultural expectation that family should provide end of life care may lead to a reluctance to engage in open discussions to facilitate the perception of good end of life care (Chan and Kayser‐Jones 2005). Similarly, a lack of trust that good care would continue at end of life had a negative impact on families and increased worry.

It is very difficult to get Chinese people to talk about death. They don't want to face or accept death. There is a lot of feeling that death is a bad omen and that if somebody dies it's a bad omen. (Chan and Kayser‐Jones 2005, 30)

He and his family were often concerned about that his tank of oxygen would run out and he would not be able to ask for help. Quote about an 81 year old Chinese man with terminal lung cancer (Chan and Kayser‐Jones 2005, 29)

Participants in another study which explored African American resident's experiences of advance care planning discussions varied from lack of opportunity to a worry about the types of interventions that would be part of end of life care.

‘My doctor never asked me, so I never brought it up.’ ‘If my doctor asks me, I'll tell him’. (Ott 2008, 120)

I don't want any tubes or anything to preserve my life. If I have to go, let me go. Let me go, if it's time to go. Don't try to save me. And I'm ready to go if today is the day. (Ott 2008, 119)

Some participants felt it was the doctors' role to make such decisions with regards to end of life care.

I haven't actually talked to him [my doctor] about it, but whatever he feels is in my best interest is OK with me. After all, he's the doctor. He has the background and the training and I feel whatever his opinion is, based on what's wrong with me is far greater than anything I could say. (Ott 2008, 120)

### 4.4 Theme 3: Person

When a person moves into a care home, they bring with them their identity and culture, which includes family, social experiences, and a set of norms. When care homes can include and adapt to these norms, it results in an enhanced quality of life for residents.

#### 4.4.1 Cultural Beliefs and Values

This sub theme includes respect for cultural heritage, religious and faith needs, and traditions such as celebrations, traditional medicine, and end of life care beliefs. These can be both a barrier and a facilitator of quality care and wellbeing.

Cultural norms of social behaviours were recognised as barriers to good quality care in several of the studies, such as forms of greetings and address to residents, a cultural unwillingness to discuss end‐ofof‐life issues and negative attitudes to forms of traditional Chinese medicine (Caldwell et al. 2014; Chan and Kayser‐Jones 2005; Haesook et al. 2014; Koehn et al. 2018; Thao et al. 2025).

Being able to celebrate festivals and religious events in a traditional way is a valued way to enhance care home resident's wellbeing. Recognition of a cultures ‘way of life’ such as the ‘Greek way’, the ‘Korean way’, the ‘Finnish way’ was a recurring theme across the literature (Heikkilä et al. 2007; Hutchinson et al. 2011; Yeboah et al. 2013; Yeboah 2015).

Social practices that focused around traditional events and cultural celebrations were deemed an important aspect of care (Heikkilä et al. 2007; Hutchinson et al. 2011; Chan and Kayser‐Jones 2005; Park et al. 2013; Thao et al. 2025; Yeboah et al. 2013). Group activities traditionally associated with care home settings such as TV, group games, and engaging with music and arts can provide enrichment or consternation, and when social experiences fit with an individual's previous culture, this can improve wellbeing (Yeboah et al. 2013; Park et al. 2013; Caldwell et al. 2014; Thao et al. 2025; Xiao et al. 2023).

I know they tried to have things like sing along[s] and concerts and all that kind of thing. … they [staff] tried their best but the last thing my mother wants is any of that. She finds it offensive. … Yes she says they insist on wheeling her. Sometimes they don't ask her. They are going to take her to the hall and my mother does not wish to go there. (Xiao et al. 2017, 63)

… for most of them, they think Bingo is the bomb, but for our ethnicity, it's—you know, we're involved in much more of the arts … like my aunt was, you know, God, reading, the arts. She considered Bingo a waste of mind, of brain cells. So how diverse are you in those social activities? (Hefele et al. 2016, 1178)

When social opportunities provided do not suit the resident's personal preferences, tension is evident.

A study conducted during the Covid‐19 pandemic highlighted the impact of pandemic restrictions on social opportunities for residents that persisted after national restrictions were relaxed. When visitors were allowed back onsite, some community areas remained out of bounds, leading to unmet preferences from some individuals (Xiao et al. 2023).

#### 4.4.2 Food

Eleven of the studies identified discussed the provision of food as an important factor in the lives of care home residents.

Then it [the second most inconvenient matter] was the food. My mother‐in‐law did not like the American food at all. So I used to bring Korean food [to the nursing home]. (Kong et al. 2010, 325)

I would like to get some soul food in the mess hall … and get Black cooks, instead of those Spanish that don't know what they're doing, don't know how to cook. Only, only thing they can do is make sandwiches. (Park et al. 2013)

In terms of cultural diversity, food plays a pivotal role in residents' physical and mental wellbeing, and has an impact on family members perceptions of care too. Having access to culturally appropriate foods and dishes within the care setting is an indicator of quality care and some studies identified issues around food being a key cause for complaint (Koehn et al. 2018; Kong et al. 2010; Park et al. 2013; Xiao et al. 2017; Girard and El Mabchour 2019). Family members will often try to bridge the gap between dietary preferences and care home food provision by bringing food from home (Thao et al. 2025, Xiao et al. 2023). Identifying issues with food may be problematic due to cultural politeness or unwillingness to raise a complaint (Xiao et al. 2017).

So, I visit my mother every day and I bring in what I've cooked at home. (Family) Me, sometimes, I bring food for my mother […] Like I said, we cook spicy […], but we can't ask to make the food spicy for everyone. Because not everyone likes that. (Girard and El Mabchour 2019, 236)

We'll always tell the family, you gotta bring food to your loved ones for them to get better. (Thao et al. 2025, 270)

Food can also support the transition of relocation into a long term setting (Yeboah et al. 2013) and when linked to familiar customs and celebrations, traditional food can aid resident's in maintaining their own ‘way of life’ (Heikkilä et al. 2007; Hutchinson et al. 2011; Thao et al. 2025). Identifying issues with food may be problematic due to cultural politeness or unwillingness to raise a complaint.

I think that Chinese, Asians are afraid to say something bad. Most likely it's like, “Good, it's quite good.” An easy example is you ask, “Is it [the food] tasty?” The reply is, “Yes, it's quite delicious.” Maybe it's not delicious, it may be too hard. They [Chinese] are afraid to voice their true opinions. (Koehn et al. 2018, 166)

An approach from staff teams that encompasses cultural humility and addresses power imbalances in the care setting, may be a strategy to overcome the challenges and help to identify individual culinary preferences (Xiao et al. 2017).

#### 4.4.3 Family

The role of the family is key throughout the process of identifying, relocating, and living in care homes for those who have families. Ten of the studies in the review included the experiences of family members in their findings (Caldwell et al. 2014; Girard and El Mabchour 2019; Hefele et al. 2016; Heikkilä et al. 2007; Koehn et al. 2018; Kong et al. 2010; Thao et al. 2025; Xiao et al. 2017, 2018, 2023).

Family members provide a link between an individual's previous life and their new life in the care home (Hutchinson et al. 2011; Koehn et al. 2018; Kong et al. 2010; Ott 2008) and this role includes facilitating communication, translating, advocating for resident's as well as maintaining family relationships.

In here we have family dinner, we tell stories to the grand children, eat … food, drink … coffee, and with a little bit of grappa, dance with the grand children. This is why my family chose this home. I didn't want to forget who I am. (Yeboah et al. 2013, 58)

The transition of care into a long term care facility can be traumatic for family members and this is often compounded by cultural expectations (Yeboah et al. 2013; Kong et al. 2010; Thao et al. 2025). For example, Hmong families typically visit in large numbers, which one study's setting found problematic due to residents sharing rooms (Thao et al. 2025).

This is what we do in … Children look after their parents when they get older and cannot look after themselves any more. You see normally the male child moves to parents’ home with his family or bring parents to live with him and his family. (Yeboah et al. 2013, 57)

We are unfilial. We already are bad children if we send our parent(s) to a nursing home. (Kong et al. 2010, 322)

Xiao et al. (2018) identified the important role that family members can have in facilitating effective cross‐cultural communication and the efficacy of adopting a collaborative approach between staff and families to developing resources to help.

… often when we have someone from a different background the families will put together a booklet of basic things, like ‘Would you like a cup of tea,’ or ‘Are you in pain?’ Along with a picture book—yeah, it's a book of a translation that we can then refer to them. (Xiao et al. 2018, 7)

Family often provide support for residents and staff to communicate when there are language differences and will provide translation in person and remotely (Thao et al. 2025; Xiao et al. 2018, 2023). Having culturally competent and bilingual staff within the care setting can vastly enhance residents having their needs and preferences met (Xiao et al. 2023).

## 5 Discussion

This systematic review identified 16 studies from around the world concerning the experiences of living in a care home from the perspective of people from ethnic minority backgrounds. Notably, the low number of studies demonstrates the dearth of research in this area, a fact previously highlighted by a similar review conducted in 2005 by Mould et al. This study employed a broad inclusion criterion to ensure experiences from both residents of care homes and their families were heard, and a range of data reviewed which provided insights into demographic, epidemiological, social, and cultural experiences of care homes. The findings are restricted to the locations of the research, which took place entirely in high‐income countries. Recent analyses indicate that despite the rapid growth of ageing populations in low‐ and middle‐income countries (LMICs), long‐term care (LTC) often remains a low policy priority (WHO 2024).

Care home services are predominantly designed to cater for the needs of residents from the mainstream culture, posing significant challenges for residents belonging to ethnic minority groups in terms of meeting their communication and care requirements (Cooper et al. 2018; Koehn et al. 2018). Recent studies highlight that care home services primarily designed for mainstream populations often face challenges in adequately meeting the communication and care needs of residents from ethnic minority groups. A 2021 systematic review identified disparities in end‐of‐life care within nursing homes, noting that racial and ethnic minority residents were less likely to engage in advanced care planning and more likely to experience inadequate pain management. The review emphasised the necessity for culturally competent care and improved communication strategies to address these disparities (Estrada et al. 2021).

The main themes to emerge from this review (Patter, Place, Person) are reflective of the multiple components that encompass good quality, culturally competent care in a care home setting. The themes were intertwined and many studies explored a combination of factors, although a few focused on one aspect of care such as food provision or living wills (Girard and El Mabchour 2019; Ott 2008). This review recognises the importance of the multi‐factorial perspectives which need to be considered in service planning and delivery. When there is greater harmony than dissonance between the themes, the better the experience of care and more needs are being met as standard.

The most prominent theme throughout the literature was Patter—communication and language, which provided context for the other themes. The challenges of language differences, norms, and cultural nuances were dominant, due to the aims of this review specifically seeking the experiences of those in underrepresented groups often living in a setting as a minority. The experiences identified in this review identify challenges from a resident or family member's perspective, something identified in previous research. A 2021 systematic review explored social conditions affecting ethnic minority residents' ability to exercise their autonomy in communication and care. The review highlighted that these residents often face significant challenges due to cultural and linguistic differences, impacting their overall well‐being (Xiao et al. 2022). The finding that communication and language are barriers to effective care is not new information and is well reiterated in the literature that explores care delivery from the staff perspective. Likupe et al.'s (2018) study of support staff and nurses working in care homes supports the identification of cultural differences, language barriers, unhelpful stereotyping, and access to practical solutions like interpreters as impeding effective care. A scoping review identified that patients from culturally and linguistically diverse backgrounds present major language barriers for nurses, which can hinder effective communication and care delivery across a range of settings and advocates for further research to investigate modes of support for nurses and care staff to work through language barriers (Gerchow et al. 2021).

The context of care varied, so some participants were living in ethno‐specific environments where language and home culture were appropriate to them; others were in the minority. When residents are underrepresented in long‐term care settings, challenges related to language differences, cultural norms, and nuanced communication are dominant factors in care experience (Chamberlain et al. 2024). Care homes have a responsibility to facilitate effective cross‐cultural communication for residents from ethnic minority groups to exercise autonomy in their care and lives. Therefore, care homes should establish and invest in resources that help cross‐cultural communication, mandate cross‐cultural care education provision for staff, and engage staff to develop rapport using a range of activities and strategies (Xiao et al. 2022).

In addition to communication, the provision of culturally appropriate food is as described by one of the included studies ‘the main concern when moving into nursing homes’ as part of care for ethnic minority residents (Xiao et al. 2023). Food is not only a basic need but also an important expression of identity and culture and providing meals that reflect the dietary preferences, religious practices, and cultural traditions of ethnic minority residents can enhance their sense of belonging and well‐being (Lillekroken et al. 2024). The UK Care Quality Commission (2023) underscores the importance of culturally appropriate care, as a quality indicator, noting that it involves being sensitive to individuals' cultural identities or heritage. Studies have shown that culturally tailored meals help residents maintain a connection to their heritage and promote positive experiences in care homes (Nemec 2020). A study examining mealtime interactions between staff and residents in a nursing home advocates for the use of culturally and linguistically adapted practices and food provision to enhance the quality of care for residents from diverse backgrounds (Liu et al. 2023). Research suggests that food‐related preferences are often overlooked in care planning, despite their significant role in residents' satisfaction and quality of life (Montayre et al. 2018). By ensuring that food practices are sensitive to cultural differences, care homes can support residents in preserving their cultural identity, which is an essential part of promoting their autonomy and improving their overall experience of care.

The themes identified around Place and Person included the multi‐ethnic environment and relationships. These experiences of living in care homes were echoed in previous reviews (Mold et al. 2005; Montayre et al. 2018), which also recognised the need to stay connected to personal lives and routines and how staff from a range of backgrounds can positively influence quality of care—particularly regarding knowledge around values, religion, beliefs and customs. A qualitative study exploring multicultural nursing home workplaces revealed that healthcare workers from minority backgrounds were more attuned to cultural diversity in daily practices compared to their majority counterparts. This awareness can enhance understanding of residents' values, religions, beliefs, and customs, thereby improving care quality (Debesay et al. 2022). This supports the recruitment and support for a culturally diverse workforce and has implications for the sector in ensuring cross‐cultural leadership and management of teams (Chen et al. 2020).

This review also identified the key role that family caregivers have in supporting the transition into care homes and enhancing quality of life. Hailu et al. (2024) focus on the vital role of family caregivers as primary providers of care for older people and the considerable challenges they face. Transition to a long‐term care setting can be a solution to these challenges for the whole family, but the sector needs awareness that family may not be adequately prepared in these roles and need support in decision‐making and provision of emotional support to those future residents. What is missing from the literature is those without that representation and support, something which may become more prominent as globalisation means more people may age in a country alone. The United Nations projects that the number of people aged 65 years and older will double in the next 25 years (Wilmoth et al. 2023) and this increase will increase the number of those who face ageing alone. The ability and choice to live alone is influenced by family support networks, and the choice of place of care is often determined by family members, who also support navigating socioeconomic factors. Access to care and support for autonomy may be reduced by those ageing alone (Reher and Requena 2018) and there is a need for policymakers to consider how this could further marginalise minoritised groups and increase inequalities in health and social care.

Care homes can go some way to address these issues by working with ethnic minority residents' families in partnership to bridge cross‐cultural communication challenges and establish a safe environment for ethnic minority residents and their families to provide feedback on the care and suggest strategies to resolve their concerns (Xiao et al. 2022). Lessons can be learned from a scoping review that evaluated the strengths and weaknesses of applying culturally adapted interventions for people from ethnic minority groups (Joo and Liu 2020). Applying these to the care home sector would mean ensuring culturally competent guidelines for care, measuring the impact of interventions and ensuring good quality training for staff. However, culturally tailored care can bring a better care experience for individuals and families, leading to better promotion of healthy lifestyles, better partnerships in care with families and an enhanced sense of belonging for those in what may be the final chapter of their life.

Maintaining a cultural connection to their previous lives is a key component of positive experiences of care homes and living in a facility embedded in that community or receiving care from staff who have a cultural affinity with residents can all support the adaptation to care homes and promote a positive living experience.

### 5.1 Strengths and Limitations

This review was limited by the sole inclusion of English language journals. This may contribute to some bias towards Western cultures, as these articles often reflect perspectives, healthcare practices, and values prevalent in English‐speaking countries, potentially overlooking the experiences and needs of non‐Western cultural groups whose literature may be underrepresented or published in other languages (NHS Centre for Reviews and Dissemination 2009). A strength, though, is the focus on the views and experiences of the people who are in receipt of care in care homes in high‐income countries. These lived experiences of those in receipt of care have been overlooked in previous literature. The use of comprehensive search strategies and multiple reviewers adds to this paper's strengths. Another key strength lies in the international scope of the papers identified which enhances the generalisability of the findings. Including only qualitative studies has provided in‐depth insights into participants' experiences; however, it is acknowledged this may limit the generalisability of the findings.

### 5.2 Implications for Future Research

This review revealed a dearth of research that seeks to identify residents' and family caregivers' experience of care homes for people from ethnic minority backgrounds. We found no UK‐based research and a complete absence of research from low‐ and middle‐income countries. With an increased need for culturally competent long‐term care to be offered across the ageing population of the world, more empirical research is required to hear the voices of these underrepresented groups about their experiences and needs. A better understanding of these concepts will lead to a more informed evaluation of care and a better ability to provide culturally competent training and education for those involved in care delivery.

## 6 Conclusion

This systematic review included 16 qualitative studies that focus on the experiences of those from ethnic minority backgrounds, living in care homes. This review identified three key themes: ‘Patter’ addresses how cross‐cultural communication skills and language barriers affect care experiences; ‘Place’ explores the impact of the care home environment, the presence of a multi‐ethnic setting, and the quality of care provided; and ‘Person’ focuses on the individual's culture, values, beliefs, food preferences, and the role of family in their care. The review sheds light on these experiences and offers valuable insights into an underrepresented area of research. The central issue identified was misalignment between care home services, often tailored towards mainstream culture's needs, and the needs of those people from ethnic minority groups. These needs are complex, interspersed with the vulnerability of being a care home resident, and encompass challenges in communication and provision of culturally appropriate care.

The review has identified the main components of culturally appropriate care, which are cultural humility in terms of communication and language, the ability of an environment to adapt to support residents' chosen way of living, and a culturally responsive approach to living well and family involvement. This has implications for the increasing number of nursing staff who develop careers in social care. This review contributes novel insights to the existing body of knowledge in research and clinical practice, identifying the need for further primary research and policy initiatives aimed at enhancing the care home provision for people from ethnic minority groups, with a focus on cultural competence, effective communication strategies, and family‐centred approaches that maintain personal connections.

## Conflicts of Interest

The authors declare no conflicts of interest.

## Supporting information


Data S1.

Data S2.

Data S3.

## Data Availability

Data sharing not applicable to this article as no datasets were generated or analysed during the current study.
