# Supplementary material for: The Experiences of People From Ethnic Minority Backgrounds Living in Care Homes—A Qualitative Systematic Review
Source: J Adv Nurs. 2025 May 19;82(3):1928–46. doi: 10.1111/jan.17060 (PMC12907587; doi:10.1111/jan.17060)
Supplement: Supplementary file 1 — Data S1. [file JAN-82-1928-s003.docx]

| **Study** | **Quality rating** | **Usefulness rating** |
| --- | --- | --- |
| Caldwell et al (2014) | 1 | 2 |
| Chan et al (2005) | 1 | 1 |
| Girard and Mabchour (2019) | 1 | 1 |
| Hefele et al (2016) | 1 | 2 |
| Heikkila et al (2007) | 1 | 1 |
| Hutchinson et al (2011) | 1 | 1 |
| Koehn et al (2018) | 1 | 1 |
| Kong et al (2010) | 1 | 1 |
| Ott (2008) | 1 | 2 |
| Park et al (2013) | 1 | 1 |
| Thao et al (2025) | 1 | 1 |
| Xiao et al (2023) | 1 | 1 |
| Xiao et al (2017) | 1 | 1 |
| Xiao et al (2018) | 1 | 1 |
| Yeboah et al (2013) | 1 | 1 |
| Yeboah et al (2015) | 1 | 2 |

**Supplementary File 1: Quality and Usefulness Rating chart (adapted from Taylor et al 2012)**
