# Supplementary material for: The Experiences of People From Ethnic Minority Backgrounds Living in Care Homes—A Qualitative Systematic Review
Source: J Adv Nurs. 2025 May 19;82(3):1928–46. doi: 10.1111/jan.17060 (PMC12907587; doi:10.1111/jan.17060)
Supplement: Supplementary file 2 — Data S2. [file JAN-82-1928-s001.docx]

*Table 2 – Search Strategy – CINAHL – Last search update 12^th^ February 2025*

| **#** | **Query** | **Limiters/Expanders** | **Results** |
| --- | --- | --- | --- |
| S18 | S14 AND S17 | Limiters - English Language Expanders - Apply equivalent subjects Search modes - Proximity | 183 |
| S17 | S15 OR S16 | Limiters - English Language Expanders - Apply equivalent subjects Search modes - Proximity | 595,892 |
| S16 | TI ( qualitative OR experience* OR perception* OR view* OR atittude* OR opinion* OR perspective* OR belief* ) OR AB ( qualitative OR experience* OR perception* OR view* OR atittude* OR opinion* OR perspective* OR belief* ) | Limiters - Publication Date: 20040101-20251231; English Language; Peer Reviewed; Research Article Expanders - Apply equivalent subjects Search modes - Proximity | 564,480 |
| S15 | (MH "Qualitative Studies+") | Limiters - Publication Date: 20040101-20251231; English Language; Peer Reviewed; Research Article Expanders - Apply equivalent subjects Search modes - Proximity | 153,555 |
| S14 | S7 AND S10 | Limiters - Publication Date: 20040101-20251231; English Language; Peer Reviewed; Research Article Expanders - Apply equivalent subjects Search modes - Proximity | 584 |
| S13 | S7 AND S10 | Limiters - Publication Date: 20040101-20251231; English Language; Peer Reviewed Expanders - Apply equivalent subjects Search modes - Proximity | 683 |
| S12 | S7 AND S10 | Limiters - Publication Date: 20040101-20251231; English Language Expanders - Apply equivalent subjects Search modes - Proximity | 709 |
| S11 | S7 AND S10 | Limiters - English Language Expanders - Apply equivalent subjects Search modes - Proximity | 849 |
| S10 | S8 OR S9 | Limiters - English Language Expanders - Apply equivalent subjects Search modes - Proximity | 2,186,408 |
| S9 | (MH "Nursing Home Residents") | Limiters - English Language Expanders - Apply equivalent subjects Search modes - Proximity | 15,539 |
| S8 | TI ( resident* OR patient* ) OR AB ( resident* OR patient* ) | Limiters - English Language Expanders - Apply equivalent subjects Search modes - Proximity | 2,180,695 |
| S7 | S3 AND S6 | Limiters - English Language Expanders - Apply equivalent subjects Search modes - Proximity | 1,230 |
| S6 | S4 OR S5 | Limiters - English Language Expanders - Apply equivalent subjects Search modes - Proximity | 56,422 |
| S5 | (MH "Nursing Homes") | Limiters - English Language Expanders - Apply equivalent subjects Search modes - Proximity | 26,022 |
| S4 | TI ( “nursing home*” OR “care home*” OR “residential home*” OR “long term care” ) OR AB ( “nursing home*” OR “care home*” OR “residential home*” OR “long term care” ) | Limiters - English Language Expanders - Apply equivalent subjects Search modes - Proximity | 47,199 |
| S3 | S1 OR S2 | Limiters - English Language Expanders - Apply equivalent subjects Search modes - Proximity | 151,310 |
| S2 | TI ( ethnic* OR “minorit* group*” OR minority OR race OR “culturally and linguistically diverse” OR CALD ) OR AB ( ethnic* OR “minorit* group*” OR minority OR race OR “culturally and linguistically diverse” OR CALD ) | Limiters - English Language Expanders - Apply equivalent subjects Search modes - Proximity | 145,975 |
| S1 | (MH "Minority Groups+") | Limiters - English Language Expanders - Apply equivalent subjects Search modes - Proximity | 15,252 |
