# Supplementary material for: The Experiences of People From Ethnic Minority Backgrounds Living in Care Homes—A Qualitative Systematic Review
Source: J Adv Nurs. 2025 May 19;82(3):1928–46. doi: 10.1111/jan.17060 (PMC12907587; doi:10.1111/jan.17060)
Supplement: Supplementary file 3 — Data S3. [file JAN-82-1928-s002.docx]

**Supplementary File 3: Example of analytical process applying Braun and Clarke’s (2022) Thematic Synthesis**

| **Extracts from Primary Studies** | **Initial Code** | **Sub-theme** | **Final Theme** |
| --- | --- | --- | --- |
| *‘But there is not many of us that can say that we speak English especially when it is necessary. When it is necessary, we find somebody here to translate’ p6*  Xiao et al 2023 | Language barriers | Communication and Language | **Theme 1: Patter** |
| *‘It is very difficult to get Chinese people to talk about death. They don’t want to face or accept death. There is a lot of feeling that death is a bad omen and that if somebody dies it’s a bad omen.’ p30*  Chan et al 2005 | Social norms of values and staff  Cultural beliefs | Cultural beliefs and values  Multi ethnic environment | **Theme 2: Place** |
| *‘I would like to get some soul food in the mess hall ... and get Black cooks, instead of those Spanish that don’t know what they’re doing, don’t know how to cook. Only, only thing they can do is make sandwiches*. ‘ p384  Park et al 2013 | Unfamiliar food,  Quality of Life | Individual’s culture  Food | **Theme 3: Person** |
